# Emerging Roles of Eosinophils and Eosinophil-Derived Lipid Mediators in the Resolution of Inflammation

**DOI:** 10.3389/fimmu.2012.00270

**Published:** 2012-08-28

**Authors:** Yosuke Isobe, Taiga Kato, Makoto Arita

**Affiliations:** ^1^Department of Health Chemistry, Graduate School of Pharmaceutical Sciences, University of TokyoTokyo, Japan; ^2^Precursory Research for Embryonic Science and Technology, Japan Science and Technology AgencySaitama, Japan

**Keywords:** resolution of inflammation, lipid mediator, eosinophils, lipidomics, lipoxygenase, resolvins

## Abstract

Acute inflammation and its resolution are essential processes for tissue protection and homeostasis. Once thought to be a passive process, the resolution of inflammation is now shown to involve active biochemical programs that enable inflamed tissues to return to homeostasis. The mechanisms by which acute inflammation is resolved are of interest, and research in recent years has uncovered new endogenous anti-inflammatory and pro-resolving lipid mediators (i.e., lipoxins, resolvins, protectin, and maresin) generated from polyunsaturated fatty acids (PUFAs). This review presents new insights into the cellular and molecular mechanisms of inflammatory resolution, especially the roles of eosinophils, and a series of omega-3 PUFA-derived anti-inflammatory lipid mediators that they generate.

## Introduction

Inflammation is a defensive response to injury and infection, but excessive or inappropriate inflammation contributes to a range of acute and chronic human diseases. Acute local inflammation in healthy individuals is self-limited and resolves by means of an active termination program (Gilroy et al., 2004; Serhan and Savill, 2005). The mechanisms that regulate the progression and resolution of inflammation remain of interest. Lipid mediator metabolomics of self-resolving inflammatory exudates recently uncovered a new family of potent anti-inflammatory and pro-resolving mediators. These include arachidonic acid (AA)-derived lipoxins, eicosapentaenoic acid (EPA)-derived E-series resolvins, docosahexaenoic acid (DHA)-derived D-series resolvins, protectin, and maresin (Bannenberg and Serhan, 2010). In inflammatory exudates, lipid mediators change in the course of acute inflammation and resolution (Levy et al., 2001; Bannenberg et al., 2005; Blaho et al., 2009; Yamada et al., 2011). In this review, we provide an overview of the novel cellular and molecular components involved in lipid mediator class switching during resolution of inflammation, especially eosinophils and the lipid mediators that they generate.

## Inflammatory Response and the Resolution of Inflammation

**Figure 1:**
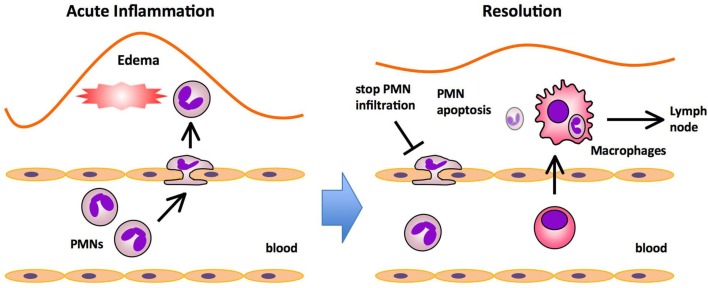
**Major processes in acute inflammation and resolution**. The initiation phase of acute inflammation is characterized by the rapid infiltration of polymorphonuclear neutrophils (PMNs) followed by the infiltration of monocytes that mature into macrophages, and edema formation in response to injury. PMNs provide the first line of immune defense by migrating to sites of injury and neutralizing invading microorganisms or noxious materials by phagocytosis. In the resolution phase, PMNs undergo apoptosis and are ingested by macrophages that emigrate rapidly from the inflamed site to the draining lymph nodes (DLNs).

Inflammation is a host defense mechanism that is characterized by the movement of serum proteins and leukocytes from the blood to the extravascular tissue. The acute inflammatory response is characterized by the initial recruitment of neutrophils, followed by the recruitment of monocytes that differentiate into macrophages (Figure 1). Many mediators coordinate the initial events of acute inflammation. Lipid mediators such as prostaglandins (PGs) and leukotrienes (LTs), cytokines, and chemokines coordinately regulate vascular permeability and leukocytes infiltration (Larsen and Henson, 1983). Once the noxious materials are removed via phagocytosis, the inflammatory reaction must be resolved to maintain homeostasis. The resolution of acute inflammation is an active process that is controlled by endogenous pro-resolving mediators. These factors switch off leukocyte trafficking to the inflamed site, reverse vasodilation, and vascular permeability, and promote the clearance of inflammatory cells, exudates, and tissue debris, thereby leading to the restoration of homeostasis to the inflamed tissue. AA-derived lipoxin A_4_ (LXA_4_) was the first PUFA-derived mediator found to have anti-inflammatory and/or pro-resolving activities (Godson et al., 2000; Serhan, 2005). Nanomolar concentrations of LXA_4_ inhibit polymorphonuclear leukocyte (PMN) entry into inflamed tissues in animal models (Colgan et al., 1993). LXA_4_ is synthesized by the actions of both 15-lipoxygenase (15-LOX) and 5-LOX, and phosphorylation of 5-LOX at S663 was recently shown to convert the enzyme to a robust 15-LOX, which can stimulate production of LXA_4_ in cells which do not express 15-LOX (Gilbert et al., 2012). Also, omega-3 fatty acids EPA and DHA are precursors of endogenous anti-inflammatory and/or pro-resolving mediators. Using an unbiased lipidomics approach and the enzymatic oxygenation of omega-3 fatty acids, Serhan and collaborators identified families of novel bioactive mediators derived from EPA and DHA. These include EPA-derived E-series resolvin (RvE1, E2; Serhan et al., 2000; Arita et al., 2005; Tjonahen et al., 2006), DHA-derived D-series resolvin (RvD1-6; Serhan et al., 2002; Sun et al., 2007; Spite et al., 2009; Chiang et al., 2012), neuroprotectin/protectin (NPD1/PD1; Hong et al., 2003; Marcheselli et al., 2003; Serhan et al., 2006), and maresin (MaR1; Serhan et al., 2009, 2012). These lipid mediators promote resolution via enhanced macrophage clearance of apoptotic PMNs, chemokines, cytokines, and microbial products (Ariel et al., 2006; Schwab et al., 2007). In the course of acute inflammation and resolution, changes of cellular composition or cell–cell interactions are accompanied by a switch of lipid mediator profiles in exudates (Serhan, 1997, 2007). The temporal switch in lipid mediator class is an active process that they underscore the ability of inflammatory cells to trigger the self-limited response of acute inflammation.

## Novel Roles of Eosinophils in Promoting the Resolution of Acute Inflammation

**Figure 2:**
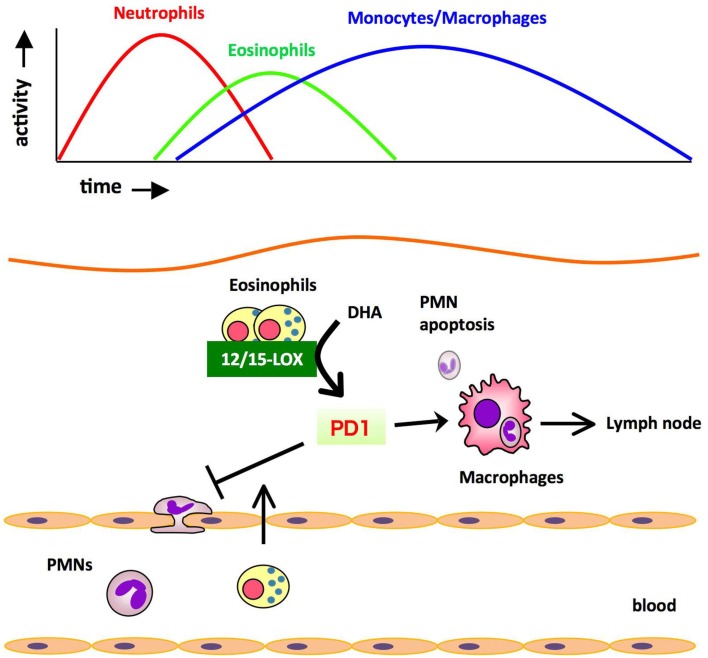
**Eosinophils are recruited to the inflamed loci and promote resolution of inflammation by producing anti-inflammatory and pro-resolving lipid mediators**. During the resolution phase of self-resolving acute peritonitis model, eosinophils are recruited to the inflammatory site, where they locally produce pro-resolving lipid mediators such as PD1 via 12/15-LOX-initiated biosynthesis. These mediators block PMN infiltration and/or promote the clearance of phagocytes carrying engulfed zymosan from the inflammatory site to DLNs.

The profile of lipid mediators during inflammation-resolution was determined with a liquid chromatography-tandem mass spectrometry (LC-MS/MS)-based lipidomic analysis. In murine zymosan-induced peritonitis, the maximal levels of 5-LOX products such as leukotriene B_4_ (LTB_4_) were observed in the initiation phase, and subsequently decreased during the resolution. In comparison, the levels of 12/15-LOX products such as protectin D1 (PD1) were low at the initiation of inflammation, then gradually increased during the resolution phase. The DHA-derived lipid mediator PD1 is biosynthesized via a 12/15-LOX-mediated pathway that converts DHA into a 10,17-dihydroxy-containing bioactive molecule (Hong et al., 2003; Marcheselli et al., 2003; Serhan et al., 2006). PD1 promotes resolution by counter regulating PMN influx and stimulating macrophage ingestion of apoptotic PMNs, and increasing phagocyte clearance into DLNs (Schwab et al., 2007). Therefore, the major cellular components of PD1 biosynthesis in the resolution phase were of interest. Recently we identified eosinophils as major PD1-producing cells in the resolution phase of zymosan-induced peritonitis (Yamada et al., 2011). Eosinophils are multifunctional leukocytes that have been implicated in the pathogenesis of numerous inflammatory processes, including parasitic infections and allergic diseases. However, the roles of eosinophils in acute inflammation and resolution are unclear. To determine the role of eosinophils in the resolution of inflammation, an anti-IL-5 monoclonal antibody (Corry et al., 1996) was administered to deplete eosinophils from mice challenged with zymosan. *In vivo* depletion of eosinophils resulted in an increased number of PMNs and a reduced number of phagocytes leaving the inflamed peritoneum to the draining lymph nodes (DLNs), both of which showed a resolution deficit. The LC-MS/MS-based lipidomic analysis revealed that the amounts of 12/15-LOX-derived mediators including PD1 were dramatically decreased in eosinophil-depleted mice, whereas the amounts of COX and 5-LOX derived products did not differ between the two groups. Adoptive transfer of wild type eosinophils, but not eosinophils from 12/15-LOX knockout mice, successfully restored the resolution phenotype. Also, administration of PD1 restored the resolution phenotype. These results indicate that eosinophils are recruited to the inflamed loci during the resolution phase, where they locally produce anti-inflammatory and pro-resolving lipid mediators such as PD1 via a 12/15-LOX-initiated biosynthetic route, which contribute to resolution (Figure 2).

Eosinophils are circulating granulocytes that typically mature in the bone marrow, and can be recruited to sites of immunological or inflammatory responses. Triggering of eosinophils by engagement of receptors for cytokines, immunoglobulins, or complement can lead to the secretion of an array of cytokines [IL-2, IL-4, IL-5, IL-10, IL-12, IL-13, IL-16, IL-18, and transforming growth factors (TGF-α/β)], chemokines (RANTES and eotaxin-1), lipid mediators [platelet-activating factor (PAF), and leukotriene C_4_ (LTC_4_)], and cytotoxic granule cationic proteins [major basic protein (MBP), eosinophil peroxidase (EPO), eosinophil cationic protein (ECP), and eosinophil-derived neurotoxin (EDN; Gleich and Adolphson, 1986; Kita, 1996]. These mediators are generally considered to be involved in the eosinophil-mediated defense against parasitic infections. Also, locally accumulated eosinophils are considered to be involved in the pathogenesis of allergic diseases such as asthma. Recently, eosinophils were shown to promote alternatively activated macrophages in an IL-4 and IL-13 dependent manner, which in turn improved glucose metabolism (Wu et al., 2011). Eosinophils in the resolution phase may modulate macrophage phenotype, and thereby promote resolution of acute inflammation.

## A Novel Eosinophil-Derived Lipid Mediator with Anti-Inflammatory Properties

**Figure 3:**
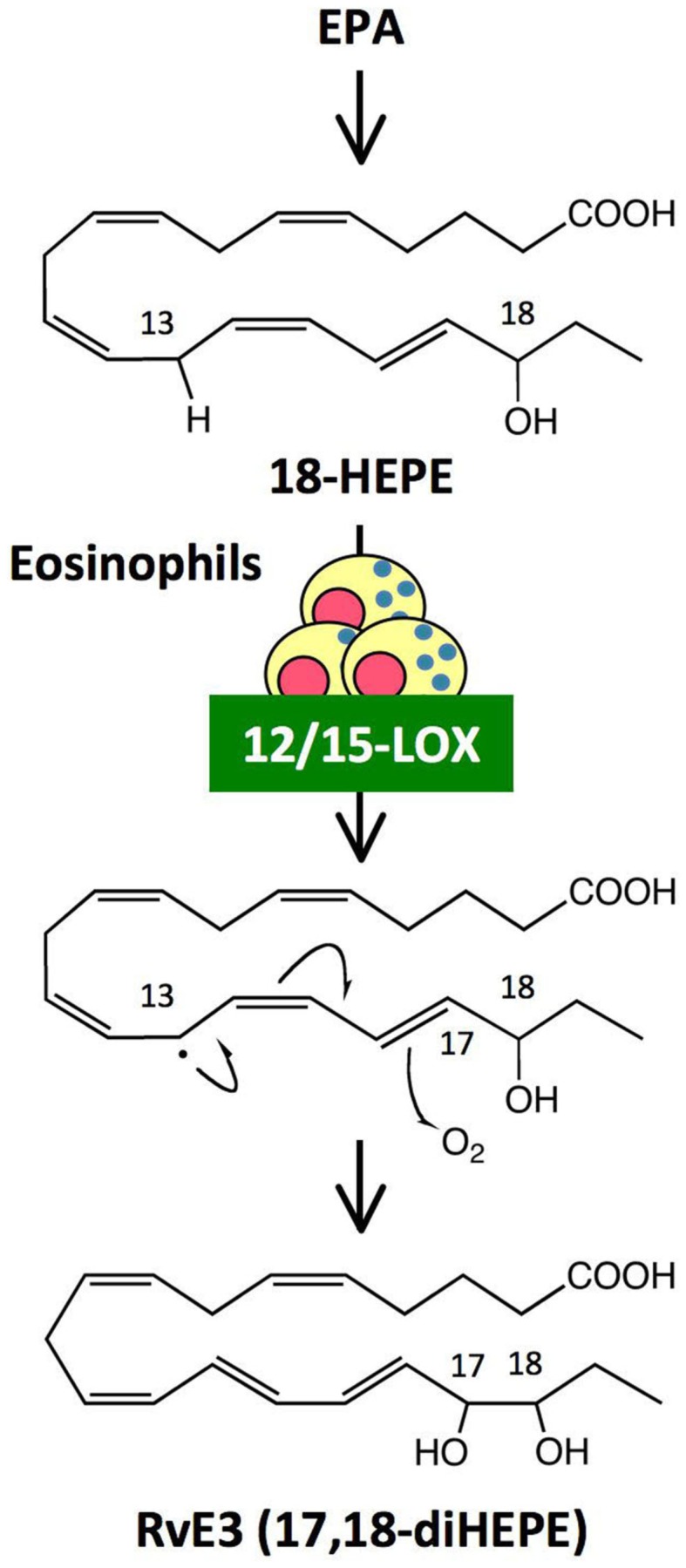
**Generation of RvE3, a novel anti-inflammatory lipid mediator, by eosinophils**. RvE3 was identified by unbiased target lipidomics. While other E-series resolvins such as RvE1 and RvE2 are biosynthesized by human PMNs via the 5-LOX pathway, RvE3 is generated by eosinophils via the 12/15-LOX pathway from a common precursor, 18-HEPE. It is likely that hydrogen abstraction from C13 by 12/15-LOX induced a stereospecific oxygen insertion at C17, leading to the formation of RvE3. RvE3 displayed a potent anti-inflammatory action by limiting PMN infiltration in zymosan-induced acute peritonitis.

Omega-3 PUFAs such as EPA and DHA are enriched in fish oils, and have beneficial effects in many inflammatory disorders including cardiovascular disease, arthritis, colitis, and asthma. Omega-3 PUFAs have been proposed to act via several mechanisms, such as by preventing conversion of omega-6 PUFA arachidonic acid to pro-inflammatory eicosanoids, and by being converted to potent anti-inflammatory mediators such as resolvins. RvE1 [5S,12R,18R-trihydroxy-eicosapentaenoic acid (EPE)] and RvE2 (5S,18R-dihydroxy-EPE) are biosynthesized by human PMNs via the 5-LOX pathway from a common precursor, 18-hydroxy eicosapentaenoic acid (18-HEPE; Serhan et al., 2000; Arita et al., 2005; Tjonahen et al., 2006; Oh et al., 2011). Analyses by unbiased target lipidomics using LC-MS/MS recently showed that eosinophils converted 18-HEPE into novel 8,18-dihydroxy-EPE (8,18-diHEPE), 11,18-diHEPE, 12,18-diHEPE, and 17,18-diHEPE (Isobe et al., 2012). Among those, 17,18-diHEPE, termed RvE3, displayed potent anti-inflammatory activity by blocking PMN infiltration in acute peritonitis. Unlike RvE1 and E2, both of which are biosynthesized by PMNs via the 5-LOX pathway, RvE3is biosynthesized via the 12/15-LOX pathway, which is highly expressed in eosinophils (Isobe et al., 2012; Figure 3). As mentioned above, eosinophils are recruited to the inflamed loci and promote resolution of inflammation. Therefore, RvE3 may, at least in part, contribute to the eosinophils’ function to regulate acute inflammation and resolution. 12/15-LOX is also expressed in tissue resident macrophages, dendritic cells, mast cells, and airway epithelial cells (Kühn and O’Donnell, 2006). 12/15-LOX deficiency leads to progressive atherosclerosis (Merched et al., 2008), exacerbation of arthritis, and inflammatory joint destruction (Krönke et al., 2009), reduced corneal re-epithelialization (Gronert et al., 2005), and a decline of self tolerance (Uderhardt et al., 2012). Cells expressing 12/15-LOX might be involved in regulating inflammatory responses by locally producing lipid mediators such as RvE3.

## Perspectives

**Figure 4:**
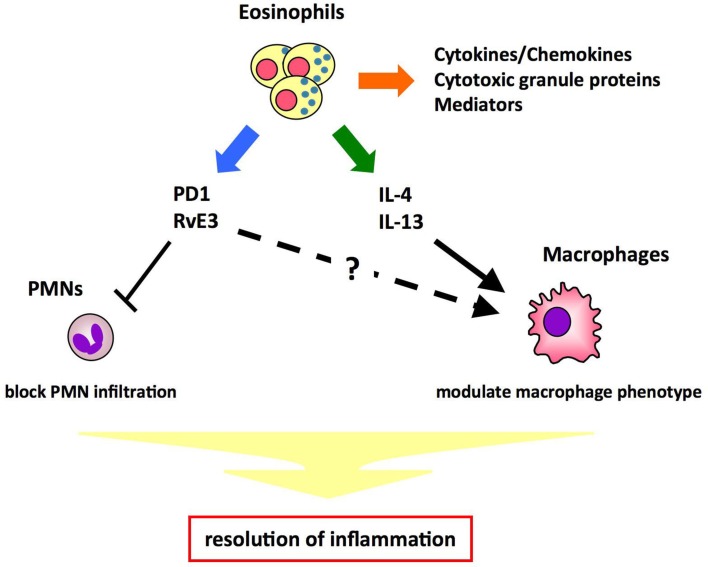
**Proposed mechanisms of eosinophils’ pro-resolving function**. Eosinophils play a role in host defense by releasing cytotoxic granule proteins, cytokines/chemokines, and mediators. In addition, eosinophils maintain metabolic homeostasis by promoting alternative macrophage activation in an IL-4- and IL-13- dependent manner. Thus, it is proposed that eosinophils promote resolution of inflammation by blocking PMN infiltration and/or modulating macrophage phenotype through cytokines (e.g., IL-4, IL-13) and/or lipid mediators (e.g., PD1, RvE3).

Eosinophils are known to be involved in allergic diseases and host protection against parasites through the release of cytokines/chemokines, mediators, and cytotoxic products. Here we provide the first evidence that eosinophils act as specific pro-resolving cells that are recruited and switched on during the resolution phase of acute peritonitis. Resolution of inflammation is a highly regulated and coordinated process that involves the suppression of PMN migration, macrophage recruitment, phagocytosis and clearance of apoptotic cells, and tissue debris. In innate immune responses, the macrophage phenotype is critical in determining whether the inflamed site resolves or progresses to chronic inflammation. Resolution phase macrophages express markers such as mannose receptor (MMR) and CD36, which are typical of alternatively activated macrophages (Fernandez-Boyanapalli et al., 2010). A recent study indicates that eosinophils promote alternative macrophage activation in an IL-4 and IL-13 dependent manner (Wu et al., 2011). Therefore, it is likely that eosinophils promote resolution of inflammation by blocking PMN infiltration and/or modulating macrophage phenotype through cytokines and/or lipid mediators (Figure 4). Detailed characterization of eosinophils in the resolution phase will provide insights into the molecular mechanisms for resolution of inflammation.

Failure of acute inflammation to adequately resolve might contribute to the development of chronic inflammation and tissue dysfunction. Indeed, some of the most common and difficult to treat diseases are linked to excessive, uncontrollable, or chronic inflammation, including cardiovascular disease, rheumatoid arthritis, periodontal disease, asthma, diabetes, and inflammatory bowel disease (IBD), as well as neurological disorders such as Alzheimer’s disease and age-related macular degeneration (AMD). The studies summarized here raise the possibility that modulating eosinophil number and function, or using endogenous mediators released from eosinophils in the resolution phase could provide novel therapies for many inflammatory diseases.

## Conflict of Interest Statement

The authors declare that the research was conducted in the absence of any commercial or financial relationships that could be construed as a potential conflict of interest.
